# A Siamese Network-Based Method for Improving the Performance of Sleep Staging with Single-Channel EEG

**DOI:** 10.3390/biomedicines11020327

**Published:** 2023-01-24

**Authors:** Yuyang You, Xiaoyu Guo, Zhihong Yang, Wenjing Shan

**Affiliations:** 1 School of Automation, Beijing Institute of Technology, Beijing 100081, China; 2 Institute of Medicinal Plant Development, Chinese Academy of Medical Sciences and Peking Union Medical College, Beijing 100193, China

**Keywords:** distance metrics, Siamese network, single-channel electroencephalogram, sleep stage classification

## Abstract

Sleep staging is of critical significance to the diagnosis of sleep disorders, and the electroencephalogram (EEG), which is used for monitoring brain activity, is commonly employed in sleep staging. In this paper, we propose a novel method for improving the performance of sleep staging models based on Siamese networks, based on single-channel EEG. Our proposed method consists of a Siamese network architecture and a redesigned loss with distance metrics. Two encoders are used in the Siamese network to generate latent features of the EEG epochs, and the contrastive loss, which is also a distance metric, is used to compare the similarity or differences between EEG epochs from the same or different sleep stages. We evaluated our method on single-channel EEGs from different channels (Fpz-Cz and F4-EOG (left)) from two public datasets SleepEDF and MASS-SS3 and achieved the overall accuracies MF1 and Cohen’s kappa coefficient of 85.2%, 78.3% and 0.79 on SleepEDF and 87.2%, 82.1% and 0.81 on MASS-SS3. The results show that our method can significantly improve the performance of sleep staging models and outperform the state-of-the-art sleep staging methods. The performance of our method also confirms that the features captured by Siamese networks and distance metrics are useful for sleep staging.

## 1. Introduction

The importance of sleep to the physical and mental health of humans cannot be overstated [1]. A growing number of people suffer from sleep disorders such as insomnia, sleep apnea syndrome and narcolepsy [2]. In order to measure sleep quality and evaluate sleep-related diseases, sleep monitoring and analysis are becoming increasingly relevant [3].

The quality of sleep is evaluated by sleep experts using polysomnograms (PSGs), which include electroencephalograms (EEGs), electrooculograms (EOGs), electromyograms (EMGs), and electrocardiograms (ECGs). In order to evaluate sleep quality, sleep staging segments PSG into 30-second epochs, and then categorizes the epochs into different sleep stages based on different sleep scoring standards, including the American Academy of Sleep Medicine (AASM) [4] and Rechtschaffen and Kales (R&K) [5]. The sleep stages are divided into awake (W), non-rapid eye movement (NREM) and rapid eye movement (REM). NREM stage is further divided into stages S1, S2, S3, S4 according to R&K and stages N1, N2, N3 according to AASM. For a long time, sleep staging relied on sleep experts to perform this labor-intensive and time-consuming task manually [6].

A lot of studies have focused on using machine learning models to automatically classify EEG epochs to their corresponding stages, such as support vector machine (SVM) [7] and random forest (RF) [8]. These methods should carefully design non-redundant and representative features and extract those features manually using algorithms like empirical mode decomposition [9] and wavelet transform [10]. Then, the features, instead of raw EEG signals, are sent to the machine learning models for classification. However, since the features were designed based on specific datasets, these methods may not generalize to different sleep datasets.

With the recent advancement of deep learning, various researchers have attempted to develop deep learning techniques based on EEG signals to aid in classifying sleep stages automatically. The existing deep learning models for sleep staging are primarily composed of convolutional neural networks (CNNs) and recurrent neural networks (RNNs) or attention mechanisms. [11]. The general approach to deep learning is to use CNNs to extract time and frequency information and other time-invariant features, followed by RNNs to learn temporal dependencies such as stage transition rules. The authors of [12] constructed a model using two CNNs with different filter sizes and two layers of bidirectional LSTMs. The architecture proposed in [13] begins with multi-resolution CNNs, followed by a temporal context encoder with multi-head attention.

**Figure 1:**
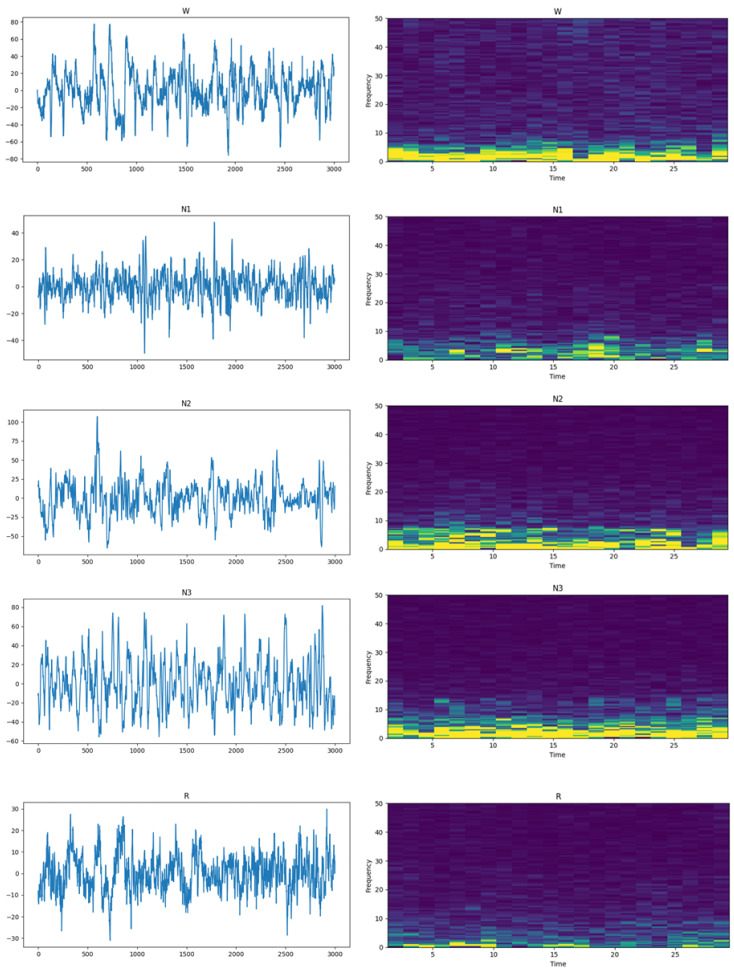
EEG epochs in the time and frequency domain. From top to bottom are stages W, N1, N2, N3 and REM. EEG epochs in the time domain are displayed on the left and the short-time Fourier transform (STFT) of EEG epochs is shown on the right. The yellow color represents higher amplitudes and the blue color indicates lower amplitudes.

The shortcomings of manually designed features can be effectively overcome by deep learning methods. However, we find that patterns representing differences or similarities between two epochs that are overlooked by deep learning models can be extracted by Siamese networks. Figure 1 shows EEG epochs in the time domain and STFTs of the signal. It can be seen from the figure that there are significant differences in EEG epochs between different sleep stages in both the time domain and frequency domain. To extract these features, we tend to design a new architecture. Siamese networks have two of the same structures which share parameters for a pair of sleep epochs’ input, and have been widely used and achieved great success in natural language processing (NLP) [14] and object detection [15]. In the field of sleep staging, Siamese networks can encode similarity and dissimilarity patterns between two epochs. The features extracted by Siamese networks are then sent to sleep staging models to improve the performance of classification models. In order to achieve better performance in sleep staging, we proposed a method based on Siamese networks, contrastive loss, Euclidean distance and max-sliced Wasserstein distance [16,17] for automatic sleep staging using single-channel EEG.

In this research, we provide a novel approach to enhance the performance of sleep staging models. The primary contributions of our study are as follows:

(1) We propose a Siamese network architecture and implement Siamese convolutional neural networks (Siamese CNNs) and Siamese autoencoders (Siamese AEs). The Siamese network architecture is used to extract features of similarity between two different epochs of EEG.
(2) To train sleep staging models with a Siamese network, we developed a new loss function. The new loss function incorporates metrics for traditional sleep staging models such as cross entropy loss and distance metrics for measuring the distance between two distributions.
(3) We evaluated our model on two public datasets, SleepEDF and MASS-SS3, and compared our results with baseline sleep staging models and some SOTA sleep staging methods. The results show that our encoder can greatly improve the accuracy of the baseline model and outperform the state-of-the-art models in sleep staging.

The rest of this paper is organized as follows. Section 2 shows the datasets and explains the methods by illustrating the architecture of Siamese AEs and Siamese CNNs, the loss function and the training process of our model. Section 3 shows the experimental results and the comparison against the baseline models. Section 4 draws the conclusion of our work.

## 2. Materials and Methods

### 2.1. Datasets

We used two different EEG channels from two public datasets, the Montreal Archive of Sleep Studies (MASS) [18] and SleepEDF [19], respectively.

SleepEDF was obtained from PhysioBank, containing two subsets, which were Sleep Cassette (SC) and Sleep Telemetry (ST). The SC subset was from a study aimed at exploring the age effects on sleep of healthy participants, while the ST subset was involved in research focusing on the effects of temazepam on sleep. Each PSG in this dataset contained two EEG channels (Fpz-Cz and Pz-Oz) with a sampling rate of 100 Hz, one chin EMG channel, one EOG channel and 1 oro-nasal respiration signal. Here, Fpz-Cz and Pz-Oz represent the positions of the electrodes. Each 30-second epoch of recordings was labeled by sleep experts based on the R&K standard. In our experiments, we utilized the Fpz-Cz EEG channel in the SC subset to train and evaluate our model. According to previous work [20,21,22,23], we merged the N3 and N4 stages into N3 and we excluded MOVEMENT and UNKNOWN.

The database MASS has 5 subsets, SS1-SS5. The subsets were arranged in accordance with their protocols for study and acquisition and the subset we utilized was SS3. Compared with SleepEDF, MASS-SS3 contained many more EEG channels. Each recording in SS3 was composed of 20 EEG channels, 3 EMG channels, 2 EOG channels and one ECG channel. The EEG and EOG recordings shared a sampling rate of 256 Hz. A notch filter of 60 Hz pre-processed the EEG and EOG recordings; then, the EEG and EOG passed the band-pass filters of 0.30–100 Hz and 0.10–100 Hz, respectively. The recordings were classified by sleep specialists into one of five stages, in accordance with the AASM standard. We selected the F4-EOG (Left) channel to train and evaluate our model. Here, F4-EOG (Left) indicates that the electrodes are placed close to the outer lower canthus of the left eye and the hairline. Furthermore, it has been found that in both the Sleep-EDF and MASS datasets, there are long periods of stage W in the recordings. However, too many or too few W stages in the training set may lead to a class imbalance problem. To avoid this issue and emphasize sleep data rather than wake data, we only used 30 min of wake periods before and after the sleep process.

Table 1 shows the number of EEG epochs of each sleep stage of the two datasets.

In SleepEDF, there were 20 participants in total, with 10 males and 10 females. The ages of participants ranged from 25 to 34, with a mean of 28.7 and a standard deviation of 2.9. There were more subjects in MASS-SS3, containing 28 males and 34 females, and a wider age range of 20 to 69. The mean and standard deviation of the ages were 42.5 and 18.9, respectively.

### 2.2. Siamese Networks

**Figure 2:**
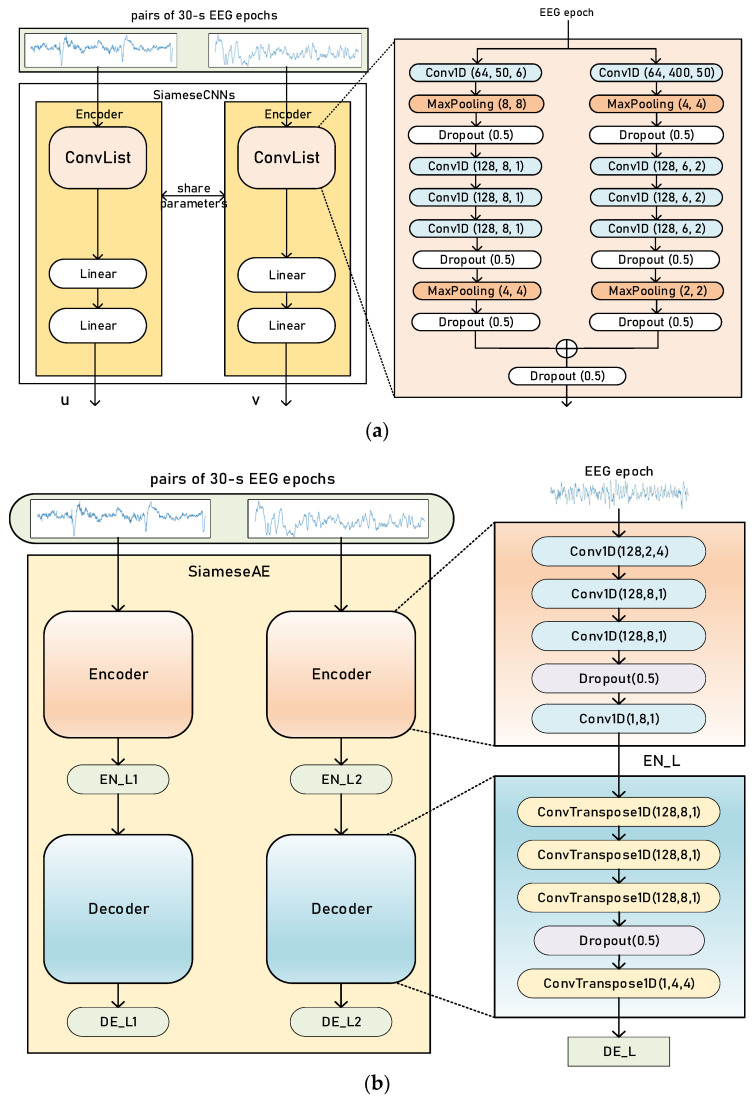
An overview of Siamese CNNs (**a**) and Siamese AEs (**b**).

We proposed two Siamese networks with different architectures. One was Siamese CNNs; the other was Siamese AEs. Figure 2a,b shows the architecture of Siamese CNNs and Siamese AEs respectively.

For Siamese CNNs, we employed two-branched CNNs to encode the input pair of EEG epochs, as shown in Figure 2a. According to previous studies [12,24], convolution with small filter sizes is better for extracting temporal information. Convolution with a larger filter size is better for extracting frequency information from EEG. Moreover, different filter sizes can also capture features from various frequency bands [13]. Therefore, the filter (kernel) sizes of the two CNN branches were different.

A pair of EEG signals with the same or different labels was the input to our Siamese CNNs. The details of EEG data pairs will be further explained in Section 2.4. Each Conv1D block was composed of 1D-convolution, batch normalization [25] and rectified linear unit activation (ReLU). The kernel size, number of filters and stride size are shown in Figure 1. The MaxPooling block shows the pooling size and the stride of the pooling layer, which downsamples the input with a max operation. The dropout layer sets neurons in the neural network to zero with a probability of 0.5.

For Siamese AEs, the encoder consists of convolutional layers and the decoder is formed of transposed convolutional layers. Transposed convolution, by mapping smaller feature maps into larger ones, is an upsampling technique, rather than the reverse of convolution. The encoder creates a latent representation *EN_L* of the input EEG, which is the output of the encoder. The decoder reconstructs the input EEG. *DE_L* is the output of the decoder.

### 2.3. Loss Functions for Measuring Similarity

We propose a novel loss function to measure the performance of the classification model as well as the Siamese network. As the Siamese network consists of two neural networks with shared parameters and architecture, which map a pair of inputs into latent features, it utilizes distance metrics to measure similarity, which is called contrastive loss [26]. The distance should be as small as possible if the input pair is obtained from the same sleep stage. In contrast, the distance should be as large as possible when the input pair comes from different sleep stages.

Defined is the distance function $D_{W}$ between the inputs $x_{1}$ and $x_{2}$ as the Euclidean distance between the outputs of a branch of the Siamese network $G_{W}$, shown as:

(1) $$D_{W}\left(x_{1},x_{2}\right)=||G_{W}\left(x_{1}\right)-G_{W}\left(x_{2}\right)|{|}_{2}$$

The contrastive loss of the Siamese network is shown in Equation (2):

(2) $$CT_{siam}\left(x_{1},x_{2}\right)=ℒ\left(W\right)=\overset{P}{\underset{i=1}{\sum }}ℒ(W,\left(y,x_{1},x_{2}{)}^{i}\right)$$

The contrastive loss is calculated by adding up the distance metric of EEG data pairs, where $\left(y,x_{1},x_{2}{)}^{i}\right)$ is the *i*-th data pair. The distance metric of a single data pair is shown in Equation (3):

(3) $$ℒ(W,\left(y,x_{1},x_{2}{)}^{i}\right)=\left(1-y\right)\frac{1}{2}{\left(D_{W}\right)}^{2}+y\frac{1}{2}{\left\{max\left(0,m-D_{W}\right)\right\}}^{2}$$

In Equation (3), *y* is the label of the EEG data pair, which represents whether the two EEG epochs belong to the same sleep stage; if the same, *y* equals 1 and if not, *y* equals 0. The hyperparameter *m* (*m* > 0) is a threshold to measure the dissimilarity of the data pair, which is determined by experiments.

The loss function of our baseline sleep staging model is multi-class cross-entropy (CE) which is defined in Equation (4):

(4) $$CE=-\frac{1}{N}\overset{K}{\underset{k=1}{\sum }}\overset{N}{\underset{i=1}{\sum }}y_{i}^{k}\log{\hat{y}}_{i}^{k}$$

Here, *N* is the number of EEG epochs and K is the number of classes. $y_{i}^{k}$ is the real label of the *i*-th EEG epoch of the class *k*. Similarly, ${\hat{y}}_{i}^{k}$ is the predicted probability of the *i*-th EEG epoch of the class *k*.

For Siamese CNNs, the loss is $Loss_{CNN}=CE_{model}+CT_{siam\left(u,v\right)\;}$, where (*u*,*v*) are the input pair of EEG epochs. Considering the encoding and reconstructing characteristics of autoencoders, the loss of our Siamese AEs has an additional component, compared with Siamese CNNs, which is the distance metric between input EEG epoch and the output of the decoder. This distance metric is composed of max-sliced Wasserstein distance (max-*W* distance) and Euclidean distance. The max-*W* distance is calculated from Equation (5):

(5) $$\max-\tilde{W_{2}}\left(u,v\right)={\left[\underset{\omega \in \Omega }{\max}W_{2}^{2}\left(u^{\omega },v^{\omega }\right)\right]}^{\frac{1}{2}}$$

Here, Ω represents the set containing all directions on unit sphere, *u* and *v* are the input distributions, and ω is an element in Ω. So, the distance metric we propose is $D\left(u,\nu \right)=||u-\nu |{|}_{2}+\max-\tilde{W_{2}}\left(u,v\right)$, where $||u-\nu |{|}_{2}$ represents the Euclidean distance between *u* and *v*. The loss of our Siamese AEs is shown in Equation (6).

(6) $$Loss_{AE}=D\left(EEG-epoch,\;DE\_L\right)+CE_{model}+CT_{siam}\left(EN_{1},EN_{2}\right)$$

In Equation (6), the EEG epoch is the input of Siamese AEs, *DE_L* is the output of the decoder, $EN_{1},EN_{2}$ are the latent features of the input EEG data pair encoded by the encoder.

### 2.4. Training and Evaluation Process

**Figure 3:**
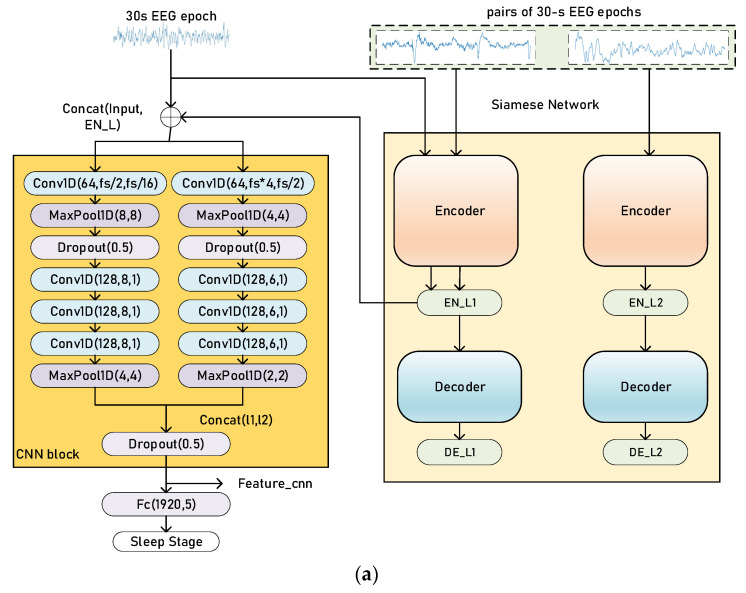

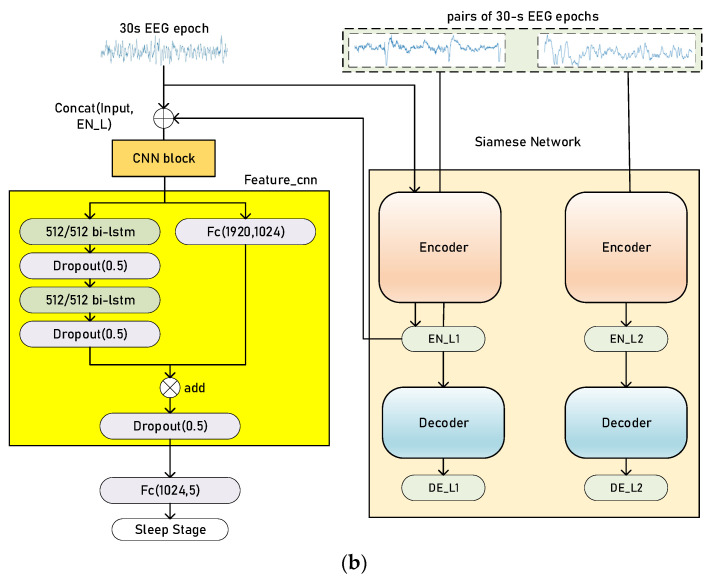
Illustration of the training process of our proposed method, pretraining (**a**) and finetuning (**b**). The Siamese network architecture is Siamese AEs. The CNN block, in orange (**a**), extracts time-invariant features, and the sequence residual block, in yellow (**b**), extracts temporal information from a sequence of EEG epochs. Concat (l1, l2) refers to the concatenation of two vectors and *fs* is the sampling rate of EEG signals. The parameters in the Conv1d blocks and MaxPooling blocks are similar to Figure 2.

There are two stages in our training process: pretraining and finetuning. The baseline sleep staging model is formed of a set of CNNs and a sequence residual learning block [27] with bidirectional long short-term memories (bi-LSTMs), which is an extension of long short-term memory (LSTM) [28,29]. The whole model (sleep staging model and Siamese network) is shown in Figure 3.

The input of Siamese networks is a pair of EEG epochs. The EEG epoch data pairs are generated by randomly selecting data pairs with the same or different labels from the training set. The label of data pairs depends on the label of each EEG epoch. If they belong to the same sleep stage, the label of data-pair is 1; otherwise, the label is 0.

During pretraining, as shown in Figure 3, a pair of EEG epochs is firstly fed into the Siamese AEs. The encoder of our Siamese AEs maps the inputs into latent features *EN_L*. Then, every 30 s, the EEG epoch is encoded by one branch of Siamese AEs, producing the latent representation to be concatenated with the original EEG epoch. The concatenated data is then fed into the sleep staging model. The basic model in the pretraining stage is the CNN block, and the output of decoder *DE_L* is used for the calculation of the loss function. Similarly, for Siamese CNNs, the latent feature vector to be concatenated is the output of the encoder. Since the loss function of Siamese CNNs does not need to reconstruct the original input, there are not any decoders in Siamese CNNs.

The sleep staging model during finetuning is based on the pretraining ones. The output of CNN block *feature_CNN* is then fed into a sequence residual learning block. The left branch of the sequence residual block is formed of bi-LSTMs with a hidden size of 512/512 and the right branch is a fully connected layer. The parameters of the Siamese network, the CNN block and the sequence residual block are updated simultaneously with different learning rates. The learning rate of the sequence residual block is larger than other blocks since they have been pretrained in the former stage.

During testing and evaluation, we used one branch of the Siamese network, since the parameters of the two branches are shared. We applied k-fold cross-validation to evaluate our Siamese network. For the two datasets involved in our experiments, k equals 20 and 31 for SleepEDF and MASS-SS3, respectively.

## 3. Results

### 3.1. Experimental Setup and Evaluation Metrics

We implemented our model with the Pytorch 1.10.0 toolkit and trained the model on a GeForce RTX 3090 GPU. We utilized the Adam optimizer [30] with an initial learning rate of 10^−3^ and a weight decay of 10^−3^ in the first training stage. The Siamese network and the CNN block of the classification model are pretrained for 100 epochs. Then, in the finetuning stage, the model is trained for 200 epochs and the learning rate of the pretrained layers is modified to 10^−6^. The learning rate of the sequence residual learning block is the same as pretraining, which is 10^−3^. The model is trained with a batch size of 32 during pretraining. In the finetuning stage, the batch size is set to 16 and the sequence length is set to 25. The threshold *m* in the contrastive loss function is set to 7400.

We evaluate the performance of our method using precision (PR), recall (RE), per-class F1-score (F1), overall accuracy (ACC), macro-averaging F1-score (MF1) and Cohen’s kappa coefficient (k) [31]. The per-class metrics $PR_{c},RE_{c},F1_{c}$ are calculated as follows:

(7) $$PR_{c}=\frac{TP_{c}}{TP_{c}+FP_{c}}$$

(8) $$RE_{c}=\frac{TP_{c}}{TP_{c}+FN_{c}}$$

(9) $$F1_{c}=\frac{2\times PR_{c}\times RE_{c}}{PR_{c}+RE_{c}}$$

Here, $TP_{c}$, $FP_{c}$, *TN_c_* and $FN_{c}$ refer to the true positives, false negatives, true negatives and false negatives of class c. When calculating per-class metrics, the positive class is the current class, and the negative class is defined as the combination of other classes.

The overall metrics *ACC, MF*1 and *kappa (k)* are defined as follows:

(10) $$ACC=\frac{\sum_{c=1}^{C}TPc}{N}$$

(11) $$MF1=\frac{\sum_{c=1}^{C}F1c}{C}$$

(12) $$P_{e}=\sum_{c=1}^{C}\frac{TP_{c}+FP_{c}}{N}\times \frac{TP_{c}+FN_{c}}{N}$$

(13) $$kappa=\frac{ACC-P_{e}}{1-P_{e}}$$

In the equations above, *C* is the number of classes which equals 5 according to the AASM manual, while *N* is the number of epochs in the test set.

### 3.2. Classification Performance

Table 2, Table 3 and Table 4 shows the per-class metrics and confusion matrices of our model on Fpz-Cz channel in SleepEDF and F4-EOG (Left) channel in MASS-SS3.

Since we evaluated our method using k-fold evaluation, we added up the metrics of each fold to calculate the confusion matrices. Each row represents the number of EEG epochs classified by humans, and each column indicates the number of EEG epochs classified by our model. We show the per-class performance (PR, RE, F1) of our models in the last three columns of the table.

In view of the better overall performance of Siamese AEs, we further evaluated Siamese AEs on dataset MASS-SS3. It can be seen from the confusion matrices that our model works well on both datasets, except for stage N1. The precisions of other stages are all around 85% while the precision of stage N1 is around 55% in SleepEDF and around 65% in MASS-SS3. The lack of EEG epochs results in the lower performance of existing sleep staging models in stage N1. Comparing the performance of Siamese CNNs and Siamese AEs on SleepEDF, Siamese AEs performs better on stages N2, N3, REM and Siamese CNNs performs better on stages W and N1. In comparison to SleepEDF, the model achieves a better result on the dataset MASS-SS3.

### 3.3. Comparison with State-of-the-Art Models

We compared our model with some other sleep staging methods and discussed the performance of our Siamese CNNs and Siamese AEs. Table 5 shows the result of our method comparing with other state-of-the-art methods.

Our baseline sleep staging network is DeepSleepNet (DSN); therefore, we compare our results with DSN first. Our Siamese CNNs and Siamese AEs both improved the performance of DSN, with increases in overall accuracy of 3% and 3.3%, respectively. The MF1 and kappa of our method also outperformed DSN on SleepEDF. To further prove that our method is effective for sleep staging, we evaluated our model on MASS-SS3 and compared the results with DSN. There was a 1% improvement in the results. Moreover, the performance of our method outperforms other SOTA sleep staging methods. Our Siamese AEs achieved the highest overall metrics among the sleep staging methods mentioned above.

Comparing our Siamese CNNs and Siamese AEs, the overall accuracy of Siamese AEs is higher than Siamese CNNs on SleepEDF, but the training time is longer than Siamese CNNs. Siamese AEs have an extra component to the loss function, so this extra constraint makes computations more complex and results in higher performance.

## 4. Conclusions

A Siamese network architecture is proposed in this study for encoding the similarity between two EEG epochs of the same sleep stage. It also encodes the differences between two EEG epochs of different sleep stages. Those features are extracted by the loss function presented by us, which is composed of distance metrics and cross entropy. Distance metrics are used to measure the distance between the distributions of two EEG epochs, and cross entropy is used to classify sleep stages. We also implemented Siamese CNNs and Siamese AEs and evaluated our method on SleepEDF and MASS-SS3. In our experiment, we used the DeepSleepNet (DSN) sleep staging model as a baseline. The results showed that the Siamese architecture proposed by us not only significantly improved the performance of DSN, but also outperformed the SOTA methods in sleep staging. In our future work, we will focus on applying our method to sequence-to-sequence models to improve the performance.

## Figures and Tables

**Table 1:** The number of samples of the two datasets involved in our work.

Dataset	W	N1	N2	N3	REM	Total
SleepEDF	7927	2804	17,799	5703	7717	41,950
MASS-SS3	6056	4598	28,311	7398	10,205	56,568

**Table 2:** Confusion matrix and per-class metrics of applying Siamese CNNs to the baseline sleep staging model on the SleepEDF dataset using 20-fold cross-validation.

	Predicted					Per-Class		
	W	N1	N2	N3	NEM	PR(%)	RE(%)	F1(%)
W	7103	394	225	49	156	87.5	89.6	88.5
N1	488	928	889	14	487	55.3	33.1	41.4
N2	330	234	15751	722	762	87	88.4	87.7
N3	26	2	706	4952	17	85.8	86.8	86.3
REM	170	120	536	35	6856	82.8	88.8	85.7

**Table 3:** Confusion matrix and per-class metrics of applying Siamese AEs to the baseline sleep staging model on the SleepEDF dataset, using 20-fold cross-validation.

	Predicted					Per-Class		
	W	N1	N2	N3	NEM	PR(%)	RE(%)	F1(%)
W	7245	370	166	39	107	85.8	91.4	88.5
N1	534	929	884	20	437	55.6	33.1	41.5
N2	435	257	15,778	718	611	87.2	88.7	87.9
N3	32	1	635	5021	14	86.5	88	87.2
REM	200	113	642	10	6752	85.2	87.5	86.4

**Table 4:** Confusion matrix and per-class metrics of applying Siamese AEs to the baseline sleep staging model on the MASS-SS3 dataset, using 31-fold cross-validation.

	Predicted					Per-Class		
	W	N1	N2	N3	NEM	PR(%)	RE(%)	F1(%)
W	5501	328	92	11	124	87.1	90.8	88.9
N1	563	2344	808	8	875	64.5	50.9	56.9
N2	163	650	25,906	786	806	91.1	91.5	91.3
N3	6	3	1326	6062	1	88.2	81.9	85.0
REM	85	311	308	3	9498	84.0	93.1	88.3

**Table 5:** Comparison of our method with other state-of-the-art (SOTA) sleep staging methods. The hyphen in the table represents that the value is unavailable.

Dataset	Method	Per-Class F1-Score					Overall Metrics		
		W	N1	N2	N3	REM	Accuracy	MF1	κ
SleepEDF-20	DeepSleepNet [12]	86.7	45.5	85.1	83.3	82.6	81.9	76.6	0.76
	SleepEEGNet [32]	89.4	44.4	84.7	84.6	79.6	81.5	76.6	0.75
	ResnetLSTM [33]	86.5	28.4	87.7	89.8	76.2	82.5	73.7	0.76
	MultitaskCNN [23]	87.9	33.5	87.5	85.8	80.3	83.1	75.0	0.77
	AttnSleep [13]	89.7	42.6	88.8	90.2	79.0	84.4	78.1	0.79
	Siamese CNNs (Ours)	88.5	41.4	87.7	86.3	85.7	84.9	78.0	0.79
	Siamese AEs (Ours)	91.4	33.1	88.7	88.0	87.5	85.2	78.3	0.79
MASS-SS3	DeepSleepNet [12]	87.3	59.8	90.3	81.5	89.3	86.2	81.7	0.80
	MixedNet [34]	84.6	56.3	90.7	84.8	86.1	85.9	80.5	-
	SeqSleepNet [35]	-	-	-	-	-	86.5	82.4	0.80
	Siamese AEs (Ours)	90.8	50.9	91.5	81.9	93.1	87.2	82.1	0.81

## Data Availability

Link to publicly archived datasets SleepEDF: https://www.physionet.org/physiobank/database/sleep-edfx (accessed on 24 March 2021).
